# Intrinsic electrical properties of cable bacteria reveal an Arrhenius temperature dependence

**DOI:** 10.1038/s41598-020-76671-5

**Published:** 2020-11-13

**Authors:** Robin Bonné, Ji-Ling Hou, Jeroen Hustings, Koen Wouters, Mathijs Meert, Silvia Hidalgo-Martinez, Rob Cornelissen, Filippo Morini, Sofie Thijs, Jaco Vangronsveld, Roland Valcke, Bart Cleuren, Filip J. R. Meysman, Jean V. Manca

**Affiliations:** 1 X-LAB, Hasselt University, Agoralaan D, 3590 Diepenbeek, Belgium; 2 Department of Biology, University of Antwerp, Universiteitsplein 1, 2610 Wilrijk, Belgium; 3 Centre for Environmental Sciences, Hasselt University, Agoralaan D, 3590 Diepenbeek, Belgium; 4 Department of Plant Physiology, Faculty of Biology and Biotechnology, Maria Curie-Sklodowska University, Plac Marii Skłodowskiej-Curie 5, 20-400 Lublin, Poland; 5 Molecular and Physical Plant Physiology, Hasselt University, Agoralaan D, 3590 Diepenbeek, Belgium; 6 Theory Laboratory, Hasselt University, Agoralaan D, 3590 Diepenbeek, Belgium; 7 Department of Biotechnology, Delft University of Technology, Van der Maasweg 9, 2629HZ Delft, The Netherlands

**Keywords:** Microbiology, Physics

## Abstract

Filamentous cable bacteria exhibit long-range electron transport over centimetre-scale distances, which takes place in a parallel fibre structure with high electrical conductivity. Still, the underlying electron transport mechanism remains undisclosed. Here we determine the intrinsic electrical properties of the conductive fibres in cable bacteria from a material science perspective. Impedance spectroscopy provides an equivalent electrical circuit model, which demonstrates that dry cable bacteria filaments function as resistive biological wires. Temperature-dependent electrical characterization reveals that the conductivity can be described with an Arrhenius-type relation over a broad temperature range (− 195 °C to + 50 °C), demonstrating that charge transport is thermally activated with a low activation energy of 40–50 meV. Furthermore, when cable bacterium filaments are utilized as the channel in a field-effect transistor, they show n-type transport suggesting that electrons are the charge carriers. Electron mobility values are ~ 0.1 cm^2^/Vs at room temperature and display a similar Arrhenius temperature dependence as conductivity. Overall, our results demonstrate that the intrinsic electrical properties of the conductive fibres in cable bacteria are comparable to synthetic organic semiconductor materials, and so they offer promising perspectives for both fundamental studies of biological electron transport as well as applications in microbial electrochemical technologies and bioelectronics.

## Introduction

**Figure 1:**
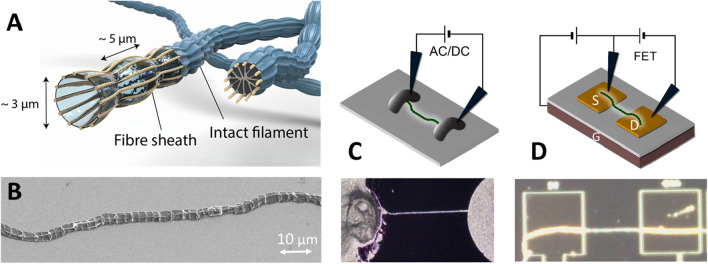
Electrical measurement set-ups for cable bacterium filaments. **(A)** A graphical representation of a cable bacterium with a set of parallel conductive fibres in the cell envelope. Filaments for electrical measurements are prepared either as intact filaments or as a fibre sheath after removal of the cytoplasm and membranes. **(B)** A SEM image of an intact cable bacterium shows the cells and ridges going along the filament. **(C)** The filament is positioned between two electrodes on a non-conductive substrate for DC or AC measurements. **(D)** In the FET measurements, two gold electrodes act as source S and drain D, while the highly n-doped silicon gate electrode G imposes the field effect at the bottom.

In 2012, a novel group of filamentous bacteria was discovered^1^, which thrive in marine and freshwater sediments^2,3^. From the analysis of the sediment chemistry, it was proposed that they can transport electrical currents over centimetre distances^1,4^. These so-called cable bacteria form unbranched chains of over 10,000 cells that vertically orient in the sediment to take advantage of the redox gradients that occur in natural sediment (Fig. 1A,B)^2^. Metabolic oxidation and reduction reactions occur in different parts of the filament, and to ensure the electrical coupling of these redox half-reactions, electrons are transported over centimetre-scale distances along the filament^1^.

Direct electrode measurements reveal that individual cable bacterium filaments can guide electrical currents over distances up to 1 cm under an externally applied potential^5^. This length scale of conduction for a single organism surpasses greatly that of other known current-producing bacteria, such as *Geobacter sulfurreducens* and *Shewanella oneidensis* MR-1. These organisms form conductive nanowires that are a few micrometres long, and act as model organisms in the field of electromicrobiology^6,7^. The conductive structures that enable the long-range transport in cable bacteria have recently been disclosed. Microscopy investigations reveal that all cells within a cable bacterium filament share a common space within the cell envelope, and that a network of parallel fibres run within this periplasmic space along the whole filament^8,9^. These periplasmic fibres comprise the primary conductive structures of cable bacteria, forming an ordered and fail-safe network^10^ with conductivities up to 79 S/cm^5^ (Fig. 1A). Nevertheless, the underlying electron transport mechanism remains currently undisclosed.

To gain fundamental insight into the long-range electron transport of cable bacteria, we have investigated the intrinsic electrical properties of individual filaments that were isolated from sediment enrichments. Using a variety of electrical characterization techniques, we studied dried intact filaments as well as so-called “fibre sheaths”, i.e. filaments from which the lipid membranes and internal cytoplasm are removed by chemical extraction, thus retaining a sheath structure that embeds the conductive fibres^5,9^ (Fig. 1A). fitted to an (RC) circuit, the parallel resistance (DC) and alternating current (AC) measurements to determine the intrinsic conductivity and the influence of contact resistances. Furthermore, the tunability of the transport was examined in a field-effect transistor set-up, which enables us to determine the charge carrier mobility. Finally, we employed the same techniques in a cryostat set-up to study the conductivity and mobility as a function of temperature.

## Results

### Cable bacteria act as resistive biological wires

In order to study the intrinsic electrical properties of cable bacteria, it is crucial to unravel the electrical equivalent circuit and the influence of contacts on the overall electrical response. Previous measurements^5^ have produced linear current/voltage (IV) curves for both individual intact filaments and fibre sheaths. Since these experiments were performed for a restricted voltage range (− 0.1 to 0.1 V), in this work we repeated them for larger voltage ranges (− 1 to 1 V and − 10 to 10 V). Measurements were conducted in a probe stage set-up with gold, silver and carbon electrodes, and to minimize degradation of the conductive structures under the influence of oxygen^5^, measurements were always performed under a nitrogen atmosphere. Regardless of the voltage range, we consistently observed the same straight IV behaviour (Fig. S1), which excludes that Schottky barriers are present at the filament/electrode interface and proving the ohmic nature of these contacts.

**Figure 2:**
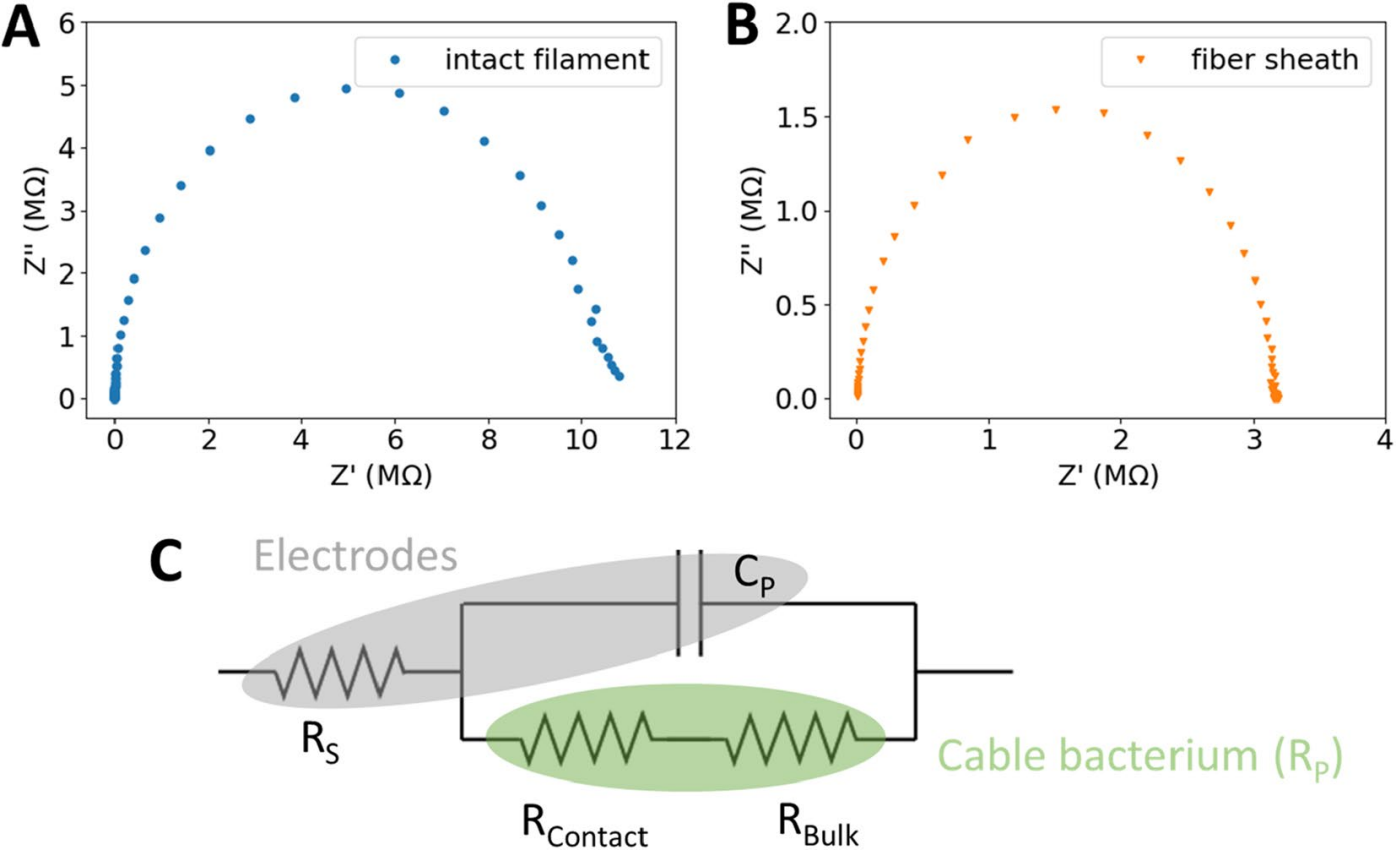
The equivalent electrical circuit for an individual cable bacterium filament probed by impedance spectroscopy. Nyquist plots of **(A)** intact filaments and **(B)** fibre sheaths show a similar single semicircle in the complex plane. **(C)** The data were described as an equivalent electrical circuit consisting of a resistor R_s_ in series with a parallel stack of a capacitor C_p_ and resistor R_p_, comprising the cable bacterium R_Bulk_ and its contact with the electrode R_Contact_.

To obtain a representative equivalent electrical circuit for cable bacteria, we performed electrical impedance spectroscopy, where an AC voltage with varying frequency (range from 1 Hz to 1 MHz; amplitude 0.1 V) is applied to a single filament in the probe stage configuration. Individual filaments were isolated from sediment enrichments and used either as an intact filament (number of samples n = 6) or as a fibre sheath (n = 4). Filaments were positioned between two gold electrodes on glass or SiO_2_ substrates with a non-conductive interspacing (100 to 500 µm) (Fig. 1C). Carbon paste was added at both ends to ensure a good electrical connection between filaments and gold electrodes (Fig. S2). All samples showed a similar response to the impedance measurements, providing a semicircle in the complex impedance plane (Fig. 2A,B). This behaviour can be described by an equivalent electrical circuit that contains two serial resistors (${R}_{s}$ and ${R}_{p}$) of which one is in parallel with a capacitor^11^ (Fig. 2C). From a reference measurement where no filament was placed between the electrodes, an equivalent circuit is obtained that does not include the resistance ${R}_{p}$ (i.e. ${R}_{p}\to \infty$), showing that the components ${R}_{s}$ and ${C}_{p}$ are inherent to the measurement setup, while ${R}_{p}$ is attributed to the filament. The equivalent electrical circuit is hence interpreted as follows: the series resistance ${R}_{s}$ represents the combination of the resistance of the measurement system wires and the resistance of the probe-electrode interface, while the capacitance ${C}_{p}$ is attributed to the capacitance of the electrodes and the measurement system. The parallel resistance ${R}_{p}$ then comprises both the bulk resistance of the cable bacterium filament ${R}_{Bulk}$ and the contact resistance between the electrodes and the filament ${R}_{Contact}$ (Fig. 2C).

Values for ${R}_{p}$ range from 0.8 MΩ to 3.6 GΩ (Table S1), corresponding with previously reported conductivity values^5^. Moreover, as expected, ${R}_{p}$ is equal in value to the total resistance of the sample measured in a subsequently performed DC measurement. Values for the other parameters were found to be ${R}_{s}=0.8\pm 1.0$ kΩ and ${C}_{p}=34\pm 15$ pF. The ratio ${{R}_{s}/R}_{p}=0.004\pm 0.008 \%$ is consistently small, which aligns with the expected low resistance of the measurement system connections. Over the broad range of experimental conditions examined, which include different filament types (intact filaments and fibre sheaths), different filament lengths, a range of filament conductivities as well as different electrode substrates, impedance results always showed a single semicircle with no distinguishable other components in parallel with the system capacitance ${C}_{p}$. In order to determine ${R}_{Contact}$, an additional DC measurement as a function of distance was performed (see Fig. S2 and Table S2), yielding a significantly smaller value compared to ${R}_{Bulk}$.

Overall, the obtained equivalent electrical circuit thus demonstrates that cable bacterium filaments can be considered as biological resistive wires with purely ohmic behaviour.

### Transistor measurements show n-type charge transport with high mobility

To determine the magnitude of the charge carrier mobility, we examined filaments in a field-effect transistor (FET) configuration (Fig. 1D), where the influence of an externally applied electric field on the conduction is evaluated. In a bottom-gate/bottom-contact FET configuration—as typically used to investigate the electrical properties of (in)organic semiconductor films—a single filament is placed across the source (S) and drain (D) electrodes separated by various channel lengths (100 to 300 µm) on top of a silicon dioxide/n-doped silicon gate (G) substrate. Since the field-effect is typically only present in a thin layer of the sample (~ 10–100 nm) near the dielectric substrate, we opted to work with fibre sheaths, for which the distance between conductive fibres and substrate is smaller than for intact bacterial filaments.

**Figure 3:**
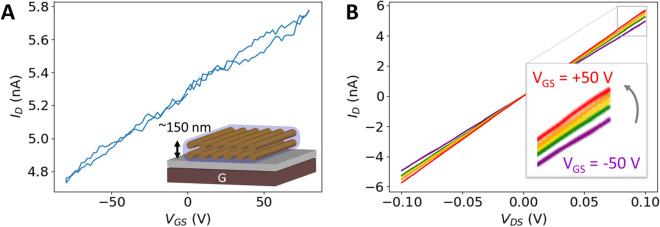
FET measurements reveal an n-type semiconductor behaviour when fibre sheaths are used as the channel. **(A)** Transfer characteristics of a fibre sheath measured at a constant ${V}_{DS}$= 0.05 V (20 °C) show a modulation of the drain current ${I}_{D}$ when the gate bias ${V}_{GS}$ is changed from 0 to 80 V to − 80 V and back to 0 V. The inset shows a fibre sheath to be a flattened ~ 150 nm double stack of fibres contained in a thin sheath. **(B)** Output characteristics of a fibre sheath under a constant gate voltage varying from − 50 to + 50 V in steps of 20 V show the slope of the current–voltage curve to change as a function of gate bias ${V}_{GS}$.

Transfer curves for a fibre sheath are shown in Fig. 3A. Here, ${I}_{D}$, ${V}_{GS}$, and ${V}_{DS}$ represent the drain current, gate-to-source voltage, and drain-to-source voltage, respectively. At zero gate bias (${V}_{GS}$ = 0) and ${V}_{DS}$ = 0.1 V, the sample shows a high off-state ${I}_{D}$, which will be further discussed later on. With increasing positive gate bias (${V}_{GS}$ > 0) at 1 V/s (other scan rates in Fig. S3), ${I}_{D}$ slightly increases (about 9% at ${V}_{GS}$ =  + 80 V). In contrast, at ${V}_{GS}$ = − 80 V, ${I}_{D}$ decreases with 9%. This indicates that the charge density at the interface between fibre sheath and dielectric increases with increasing gate voltage, consistent with n-type semiconductor behaviour where electrons are the main charge carriers. To verify this, the leakage current ${I}_{G}$ was monitored for all measurements (n = 4), which was always more than two orders of magnitude smaller (1–10 pA) than the change in ${I}_{D}$ (Fig. S4). Additionally, the output characteristics (${I}_{D}$ versus ${V}_{DS}$) were determined for ${V}_{GS}$ varying from − 50 V to + 50 V. A typical graph is given in Fig. 3B, where the gate bias modulates the linear slope (${\partial \mathrm{I}}_{\mathrm{D}}/\partial {\mathrm{V}}_{\mathrm{DS}}$) of the IV curve. The conductivity linearly increases with gate bias ${V}_{GS}$, yielding a modulation rate of 3 mS/cm per volt (Fig. S5).

Given the bias condition (${V}_{DS}\ll {V}_{GS}$), the transistor response is found to be linear over the gate voltage domain, as shown in Fig. 3A. An estimate for the mobility of the electrons can be obtained by using the formula $\mu ={(\partial I}_{D}/{\partial V}_{GS})\cdot l/(w\cdot {V}_{DS}\cdot {C}_{i})$ in the linear bias mode condition at positive gate voltage, where $l$ = 0.1–1 mm is the channel length and $w$ = 4 µm is a conservative estimate of the channel width^12^ since it corresponds to the width of the total fibre sheath. ${C}_{i}$ is the gate capacitance per unit area and can be calculated as ${C}_{i}={\varepsilon }_{r}\cdot {\varepsilon }_{0}/d$, with $d$ the substrate oxide thickness, ${\varepsilon }_{0}$ the vacuum permittivity and ${\varepsilon }_{r}$ the relative dielectric permittivity of the gate insulator. For the four fibre sheaths examined, the electron mobility was found to be in the range of 0.09–0.27 cm^2^/Vs (Table S3), which is in the same order of magnitude as many organic semiconductors^13^.

### Conduction is thermally activated with low activation energy over a wide temperature range

**Figure 4:**
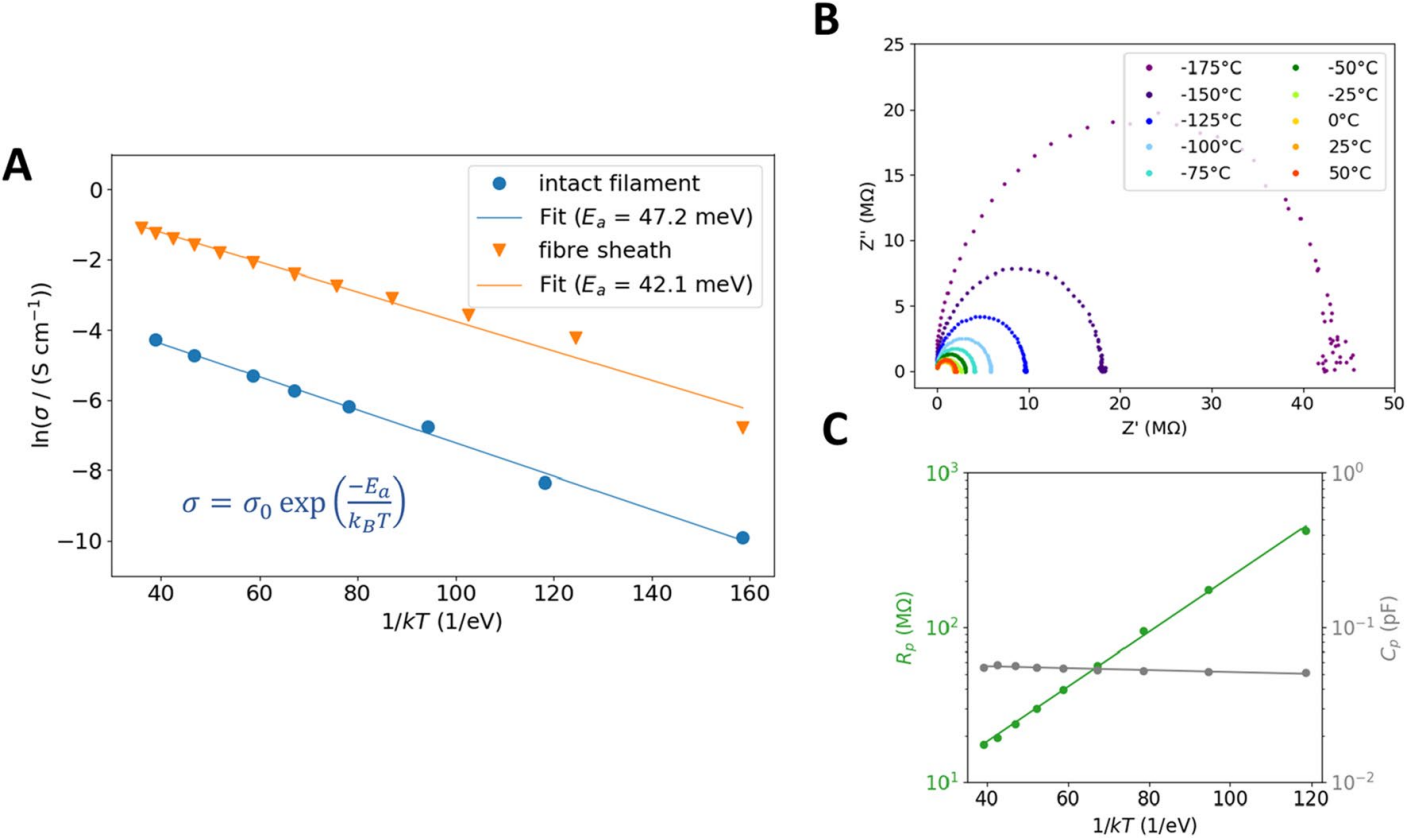
Temperature-dependent electrical characterization shows thermally activated charge transport. **(A)** The conductivity $\sigma$ of intact filaments and fibre sheaths show a linear relation with the inverted thermal energy $1/{kT}$, thus following an Arrhenius behaviour with activation energy in the range of 40–50 meV. **(B)** Independent measurements of the impedance response as a function of temperature confirm this result. The similarity in the semicircle for every temperature implies the thermal activation only to be present in the (bulk) parallel resistance. **(C)** When fitted to an (RC) circuit, the parallel resistance ${{R}}_{{p}}$ shows a similar thermal activation as found in **(A)**, while the capacitance ${C}_{{p}}$ remained constant as a function of temperature.

To further understand the charge transport mechanism^14–16^ in cable bacteria, we studied the conductivity at different temperatures for a broad temperature range in a helium atmosphere (see “Methods” section). Figure 4A shows the conductivity $\sigma$ (see “Materials and Methods”) as a function of the inverted thermal energy $1/{k}T$, for both an intact filament and a fibre sheath, when cooled down in discrete steps from + 50 °C to − 195 °C. Both filament types demonstrate a similar behaviour; the conductance decreases with decreasing temperature, thereby excluding the possibility of metal-like conduction. The activation energy ${E}_{a}$ is determined by fitting the data with the Arrhenius function $\sigma ={\sigma }_{0}\mathrm{exp}({-E}_{a}/kT)$^17^ (Fig. 4A). The fitted curves show similar slopes, indicating comparable activation energies; the differences in offsets indicate a different room temperature conductivity as observed before. Heating the samples back from − 195 to + 50 °C resulted in similar activation energy, thereby demonstrating any filament decay to be small (Fig. S6). An average of the activation energy for all samples (Table S4) results in 42.3 ± 6.5 meV for intact filaments (n = 8) and 48.4 ± 7.4 meV for fibre sheaths (n = 10)—very close to the room temperature ${k}T$ value of 25 meV and low compared to typical activation energies in the order of 500 meV for biological conductors^16^ like *S. oneidensis* nanowires^18^. This result demonstrates that electron transport in cable bacteria is thermally activated, and filaments remain conductive far beyond the natural physiological temperature range of living cable bacteria.

To verify whether the low activation energy is intrinsic, the impedance response was measured as a function of temperature in a new set of experiments. Figure 4B shows a complex plane plot for an intact filament for temperatures ranging from − 175 to + 50 °C (n = 3). Again we deduce for all temperatures a similar semicircle as shown in Fig. 4B, showing a negligible system resistance ${R}_{s}$. This time, the equivalent circuit (${C}_{p}{R}_{p}$) was fitted to the data (Table S5). The ${R}_{p}$ value agrees well with the corresponding DC value, and a similar Arrhenius behaviour is found with a corresponding activation energy ${E}_{a}$ = 40.1 ± 5.3 meV, while ${C}_{p}$ was found to be constant over the whole temperature range (Fig. 4C).

### Temperature-dependent FET measurements show a similar thermally activated mobility

**Figure 5:**
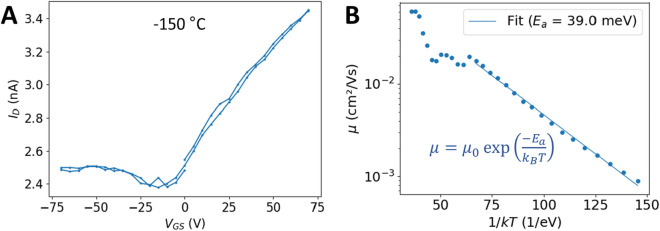
The electron mobility of the conductive structures in cable bacteria is thermally activated. **(A)** A transfer characteristic at lower temperature (at a constant ${V}_{DS}$ = 0.5 V) indicates the n-type effect is more prominent at lower temperatures. Calculated from transfer curves at different temperatures, **(B)** the mobility is plotted as a function of temperature to reveal that the electron mobility is thermally activated, following an Arrhenius relationship for temperatures below -100 °C.

In order to further unravel the electron transport mechanism, the FET characteristics are likewise studied as a function of temperature for the same range of − 195 °C to + 50 °C, with smaller increments. Fibre sheath samples were prepared as before and laid on interdigitated gold electrodes (10 lines, interspacing 20 µm) to enhance the current signal at low temperatures. For a series of 30 different temperatures, a transfer curve is made. As shown in Fig. 5A, the transfer curves at low temperature more resemble a classical n-type FET behaviour as compared to room temperature (Fig. 3A), with a higher change in ${I}_{D}$ at positive gate voltages and almost no effect at negative gate voltages.

The mobility was calculated from a fit over the positive gate voltages, again using the linear mode bias condition. In Fig. 5B the calculated mobility is plotted as a function of temperature. At temperatures above − 100 °C (I.E. the left part of the graph), the retrieved mobility values show more variation, which is attributed to a less pronounced transistor response at those temperatures (see also the “Discussion”). An Arrhenius behaviour becomes apparent over the temperature range of − 195 °C to − 100 °C. When fitting the data to the Arrhenius relationship $$\mu ={\mu }_{0}\mathrm{exp}({E}_{a}/{k}
T)$$, similar activation energy for the mobility as for the conductivity can be determined. Averaged over n = 3 measurements (Table S6), the activation energy for the electron mobility is 36 ± 5 meV, compared to a value for the activation energy of conductivity of 50 ± 2 meV, measured on the same samples.

## Discussion

In this work, we report the intrinsic electrical properties of dry cable bacterium filaments with different characterization techniques. Using electrical impedance spectroscopy, we found a single semicircle in the complex plane, indicating that cable bacteria can be considered as biological electrical wires with ohmic contacts. These results are in line with a theoretical impedance analysis for transport in stochastic systems^19^, but also correspond to *Geobacter* nanowires^20,21^ with the exclusion of an ionic component to the overall conductivity.

Alongside a high electrical conductivity (> 10 S/cm;^6^ and this work), our results demonstrate that the conductive fibres in the cell envelope of cable bacteria display high electron mobility (10^–1^ cm^2^/Vs) for a biological material. These values are similar in magnitude to organic semiconducting nanowires^13,22,23^ like P3HT^24^ and PEDOT:PSS^25^ and close to the charge carrier mobility in amorphous silicon (1 cm^2^/Vs)^22^. Furthermore, the estimated charge carrier mobility is higher than that of nanowires from *G. sulfurreducens* (10^–4^ to 10^–2^ cm^2^/Vs)^26,27^ and comparable to the hole mobility of the conductive structures of *S. oneidensis* (10^–1^ cm^2^/Vs)^28^. The promising values for the mobility for the conductive structures in cable bacteria make them an interesting candidate material in the search for organic and biological alternatives to classical semiconductors. Furthermore, cable bacteria show a transistor response that is detectable over a broad voltage range, though with a smaller effect as compared to *Geobacter*^20^ and *Shewanella* nanowires^28^, but becoming more apparent at lower temperatures. This limited tunability may be at least in part due to the particular geometric configuration of the fibre sheaths examined here. The conductive fibres are embedded in a non-conductive matrix, and only the bottom layer of the ~ 120 nm double stacked fibres is expected to be influenced by the gate electric field^9^, while the upper layer will act as a temperature dependent conductive pathway, which will disturb the transistor behaviour at higher temperatures (Fig. 4A, inset). For individually isolated fibres, we expect a higher tunability.

Our temperature-dependent experiments reveal that the conductivity and electron mobility is thermally activated and can be described by an Arrhenius relation with an activation energy of around 45 meV. Arrhenius behaviour has also been found for thin films of proteins and peptides placed between planar electrodes^15,16^, as well as in inorganic nanowires with surface defects^29,30^, and is commonly attributed to (multistep) hopping transport. However, where a multistep hopping transport is proposed for *S. oneidensis* nanowires^6^, the charge transport mechanism in *G. sulfurreducens* nanowires remains unclear and under debate^21,31–33^. Future structural and electrical studies are needed to elucidate the electrical transport mechanism inside the periplasmic fibres of cable bacteria and distinguish between multistep hopping^26,33^, variable range hopping^34^, coherence assistant hopping, or other mechanisms^30,35^.

The electrical properties of cable bacteria described here offer new perspectives not only for fundamental studies, but also for technological applications. Our observations that cable bacteria can function as electrical interconnections with low contact resistance, as well as active electrical channels in FETs, show that they can be envisioned as suitable future materials for the emerging field of bioelectronics^36^, including visionary technologies such as biodegradable electronics. The reported intrinsic electrical properties, together with the long-range electron transport and the wide temperature range of operation, are unique assets to envisage cable bacteria for these future electronic applications.

## Materials and methods

### Sample preparation

Cable bacteria were enriched in natural sediment cores incubated in oxygenated seawater, as described previously^3^. Sediment was collected at Rattekaai (Oosterschelde, The Netherlands). Single filaments of cable bacteria were picked from the sediment enrichment, as described previously^9^. Filaments were washed at least six times in MilliQ water to remove sediment debris, thus providing so-called “intact filaments”. Any excess of water was removed with a pipette and the sample was left to dry. Overall, about 5 min passed between the picking of a filament and the start of the current measurement. Alternatively, after washes with MilliQ, filaments were exposed to a sequential extraction procedure, thus removing the cytoplasm and membranes, as described previously^9^. This provided so-called fibre sheaths. After about 45 min of preparation time, the sample was transferred onto the electrode substrate.

### AC/DC electrical measurements

For all electrical measurements, the substrate was placed at a probe station with two needle probes connecting to the two electrodes. The probe stage is housed in a nitrogen glovebox to prevent sample decay. In the DC measurements, the probe station was connected to a Keithley 2450A sourcemeter (Keithley, USA) with triax cables, driven by the multi-tool control software SweepMe, as described earlier^5^. For AC impedance measurements, the sample is probed with a VersaSTAT3F potentiostat (Ametek, USA), allowing impedance measurements in the range 1 MHz to 1 Hz or 100 mHz at bias voltage 0.1 V. These results were verified with a MFIA impedance analyser (Zurich Instruments, Switzerland) for frequencies in the range of 5 MHz to 1 Hz. Data fitting was done with both the ZSimpWin and ZView software packages (Solartron, USA). Conductivity $\sigma$ was calculated for all samples using $\sigma =Gl/A$, with $l$ the conduction length, $A$ = 0.12 µm^2^ the conductive area (about 60 fibres of 50 nm diameter)^9^ and $G=\Delta I/\Delta V$ the conductance calculated with a linear fit to the IV-diagrams.

### Field-effect transistor measurements

Field-effect measurements were done on a highly n-doped silicon wafer in a bottom-gate bottom-contact FET configuration. A 150 nm thick thermally grown silicon oxide layer served as a dielectric layer, and the bottom drain and source gold electrodes with a thickness of 50 nm of the coplanar FET were defined by optical lithography to yield a channel length of 100 µm. After the washing and extraction treatments, as mentioned above, an individual filament was placed across the source and drain contacts. Gate-source and drain-source voltages were applied by two separate Keithley 2450A sourcemeters, for which the current response was continuously monitored.

The time response of the drain current upon applying a positive gate bias depends on the time to build up the conductive channel. Therefore, in order to measure consistent transfer curves, a proper gate sweeping speed was crucial. Fig. S3 shows the transfer curves measured at a gate sweeping speed varying from 10, 2, 1, and 0.5 V/s. Using a fast scanning speed of 10 V/s, the signal of ${I}_{D}$ shows a large hysteresis. Stable ${I}_{D}$ is obtained by reducing the gate sweeping speed down to 2 V/s and 1 V/s.

### Temperature-dependent measurements

Temperature measurements were performed in two different cryostats. Initial DC measurements were performed with a commercial model OptistatDN by Oxford Instruments. The cryostat is liquid nitrogen-based, allowing the sample to be cooled down from ambient temperature to − 195 °C. Cable bacterium filaments were dropcasted on glass substrates, and carbon paste was applied to both ends to form electrical contact points. These were mounted in a double-walled cryostat using spring contacts. The inner vessel was filled with helium as exchange gas, the outer vessel by a high vacuum, maintaining a pressure around 10^–9^ bar for thermal insulation. The sample was heated to 50 °C and then cooled down to − 195 °C in steps of 25 °C. For each step, current–voltage curves were measured to obtain the conductivity $\sigma$. Cooling down was achieved by adjustment of a needle valve for the liquid nitrogen flow towards the heat-exchanger. A steady-state temperature is established by the combination of the heat exchanger and an adjacent heating element. The latter was coupled in a feedback loop to a temperature control unit, model ITC 502 by Oxford Instruments. After stabilization of the current (about 15 min), a current–voltage measurement was performed at each temperature.

For temperature-dependent AC and FET measurements, and as verification experiments of the previous set-up, a cryostat probe stage HFS350EV-PB4 with liquid nitrogen pump LNP96-S and LINK software was used (Linkam, UK). Coax outlets were coupled to the MFIA or adapted to triax cables to connect to the Keithley 2450A sourcemeters. The prepared sample is loaded on the stage and purged with nitrogen gas for 3 min. It was then cooled down to − 195 °C with liquid nitrogen in the disk underneath the sample. In steps of 25 °C or smaller, the sample is heated to 50 °C. The current was monitored to stabilize (after 1 to 5 min) before the characterization measurement was performed. For AC measurements, a bias voltage of 300 mV was applied; for FET measurements ${V}_{DS}=5 V$ was chosen to enhance the current output signal. After the measurements, all samples were studied with an optical microscope to verify if there was no degradation or damage due to cooling to low temperatures.

### Statistics

All measurements were at least performed in triplicates. The value “n” in the text symbolizes the number of samples, and averages are given $\pm$ the standard deviation.

## Supplementary information

Supplementary Information.
