# Supplementary material for: Intrinsic electrical properties of cable bacteria reveal an Arrhenius temperature dependence
Source: Sci Rep. 2020 Nov 13;10:19798. doi: 10.1038/s41598-020-76671-5 (PMC7666173; doi:10.1038/s41598-020-76671-5)
Supplement: Supplementary file 1 — Supplementary Information. [file 41598_2020_76671_MOESM1_ESM.pdf]

Supplementary Information

Intrinsic electrical properties of cable bacteria reveal an Arrhenius temperature dependence

Robin Bonné[1], Ji-Ling Hou[1], Jeroen Hustings[1], Koen Wouters[1], Mathijs Meert[1], Silvia Hidalgo-Martinez[2], Rob Cornelissen[1], Filippo Morini[1], Sofie Thijs[3], Jaco Vangronsveld[3,4] , Roland Valcke[5], Bart Cleuren[6], Filip J. R. Meysman[2,7] and Jean V. Manca[1]*

[1] X-LAB, Hasselt University, Agoralaan D, B-3590 Diepenbeek, Belgium
[2] Department of Biology, University of Antwerp, Universiteitsplein 1, B-2610 Wilrijk, Belgium
[3] Centre for Environmental Sciences, Hasselt University, Agoralaan D, B-3590 Diepenbeek, Belgium
[4] Department of Plant Physiology, Faculty of Biology and Biotechnology, Maria Curie-Sklodowska University, Plac Marii Skłodowskiej-Curie 5, 20-400 Lublin, Poland
[5] Molecular and Physical Plant Physiology, Hasselt University, Agoralaan D, B-3590 Diepenbeek, Belgium
[6] Theory Lab, Hasselt University, Agoralaan D, B-3590 Diepenbeek, Belgium
[7] Department of Biotechnology, Delft University of Technology, Van der Maasweg 9, 2629HZ Delft, The Netherlands.

* Corresponding author: jean.manca@uhasselt.be

Distance dependent measurements reveal small contact resistance

To further quantify the influence of the contact resistance we used a variation of a transmission line measurement in which we measure the conductivity as a function of distance. We placed one filament between a series of equally distanced (500 µm) gold electrodes without applying carbon paste. Then we measured the current through each pair of adjacent electrodes (X and Y) when imposing a potential of 0.1 V (Fig. S2A). A very low current was detected for the pairs 3-4 and 4-5. When measuring on the pair 3-5 however, a higher current was detected, suggesting a high contact resistance for electrode 4. When we ensured the connection with the gold electrode for each part with carbon paste, the signal drastically enhanced (Fig. S2B), showing the contact at electrode 4 was 'fixed'. This confirms the earlier stated importance of carbon paste for a good electrical connection (Meysman et al., 2019).

We now measured the current between all pairs of electrodes (n=3). At first it is striking that the current at different parts of the filament shows a high variance, showing a current between 1 and 2 that is three times higher than between 4 and 5. This might be due to a high variance in contact resistance or bulk resistance. However, when measuring the resistance e.g. between electrodes 1 and 4 this exactly equals the resistance for electrodes 1-2, 2-3 and 3-4. The same goes for all other possible combinations (Table S1). This indicates that, when measuring electrodes 1-4, around electrode 2 and 3 the current chooses the path of least resistance and flows through the gold electrode. Since 1-4 shows no additional resistance, this means that the contact resistance is negligibly small as compared to the bulk resistance. Moreover, the results show that there is a high intrinsic variability in conduction over the length of a single organism.

Fig S1: IV characteristics for cable bacteria over a large voltage range

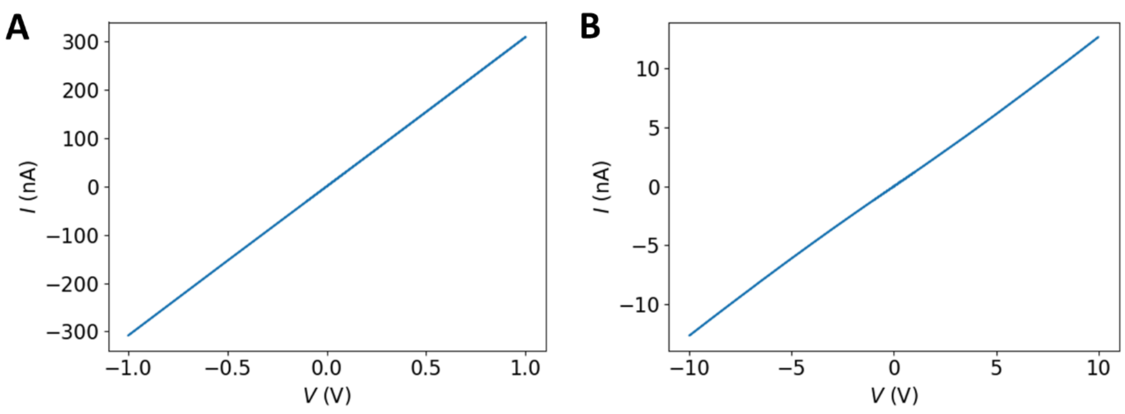

Fig. S1. IV characteristics for (A) a fibre sheath and (B) an intact filament over large voltage ranges show the contacts to be ohmic rather than any Shottkey contacts being present.

Fig S2: Distance dependent measurements reveal the contact resistance

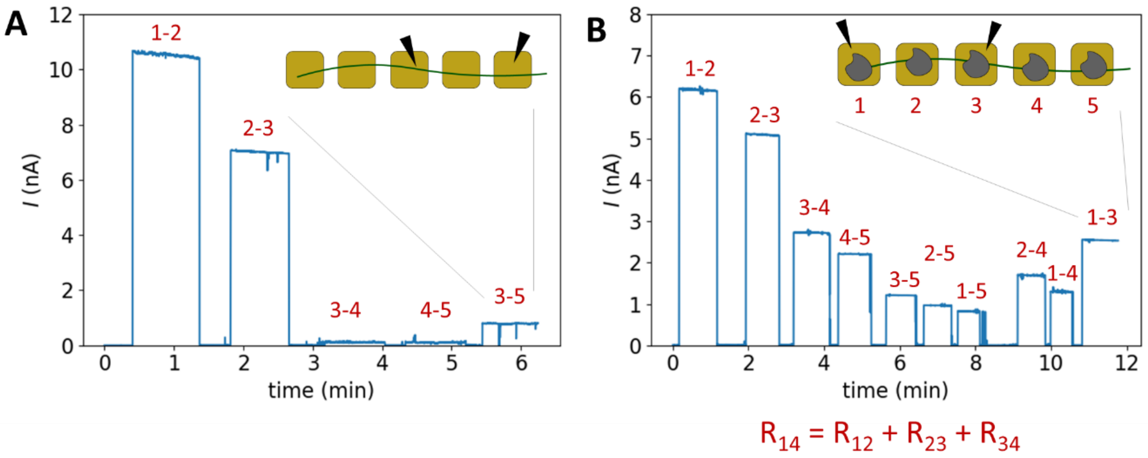

Fig. S2. For the experiment described in Fig. 2A a filament was placed on a series of equidistant electrodes to measure the current between each two electrodes. Before applying carbon paste, this result was obtained. A very low current was detected for the pairs 3-4 and 4-5. When measuring on the pair 3-5 however, a higher current was detected, suggesting a high contact resistance for electrode 4. As is seen from Fig. 2A, application of carbon paste drastically enhanced the signal and 'fixed' the contact at electrode 4.

Fig S3: FET transfer curves for different gate sweeping voltage rates

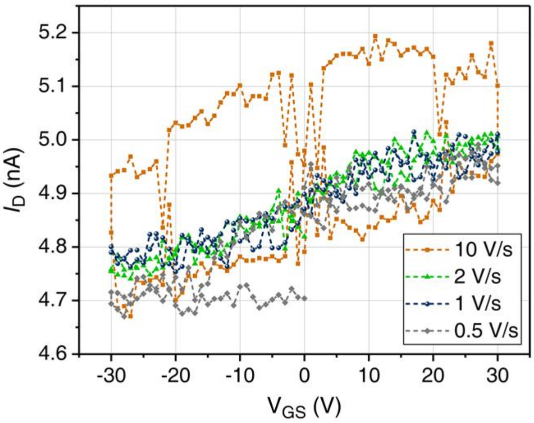

Fig. S3. Linear transfer characteristics of fibre sheath CB-FET measured at a gate sweeping speed varying from 10 V/s, 2 V/s, 1 V/s and 0.5 V/s. V_{DS} is constant at 0.05 V.

Fig S4: Low leakage currents for FET measurements

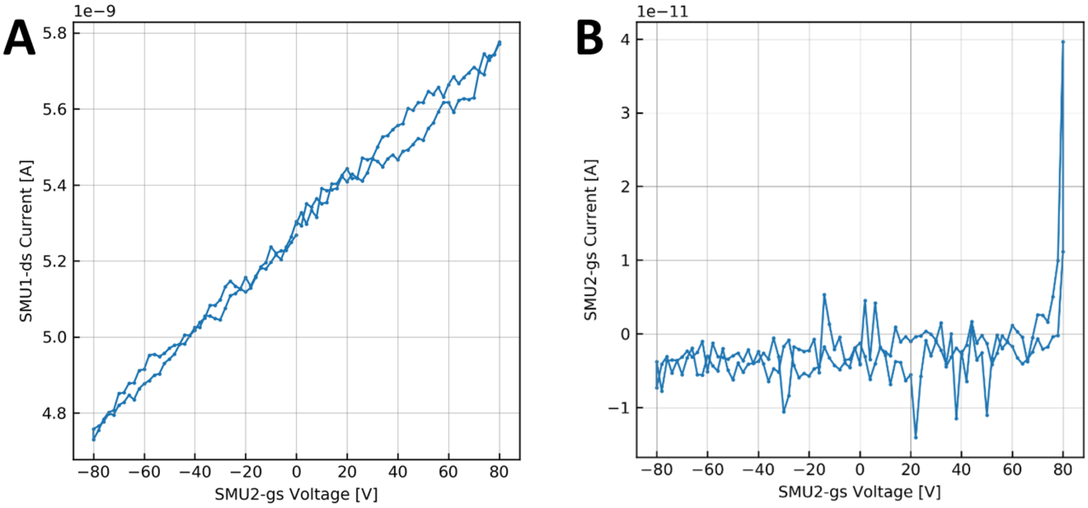

Fig. S4. For all FET measurements, (A) a transfer curve of $I_D(V_{GS})$ is always determined in parallel with a measurement of (B) the leakage current $I_G(V_{GS})$. This parasitic current was always found in the range 1 – 10 pA, or 2 orders of magnitude lower than the variation in current through the filament ΔI_D.

Fig S5: Conductivity change from FET characteristics

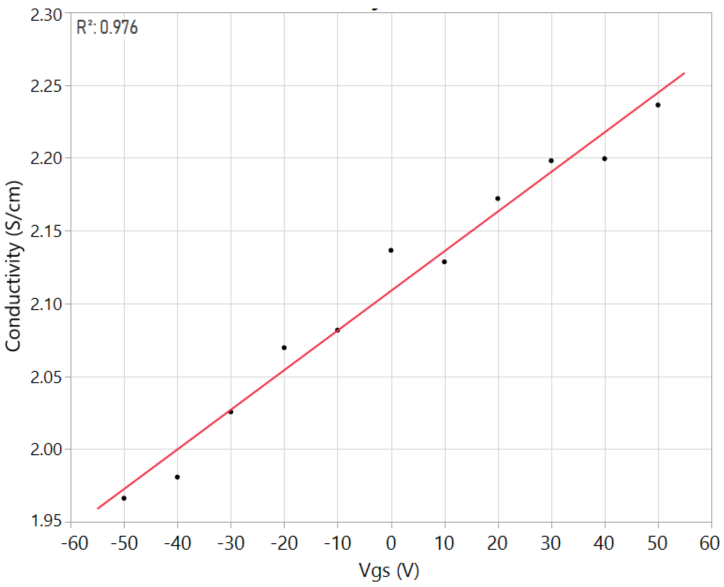

Fig. S5. Conductivity as a function of gate voltage V_{GS} shows a modulation rate of about 3 mS/cm.

Fig S6: Cooling versus heating shows the same Arrhenius behaviour

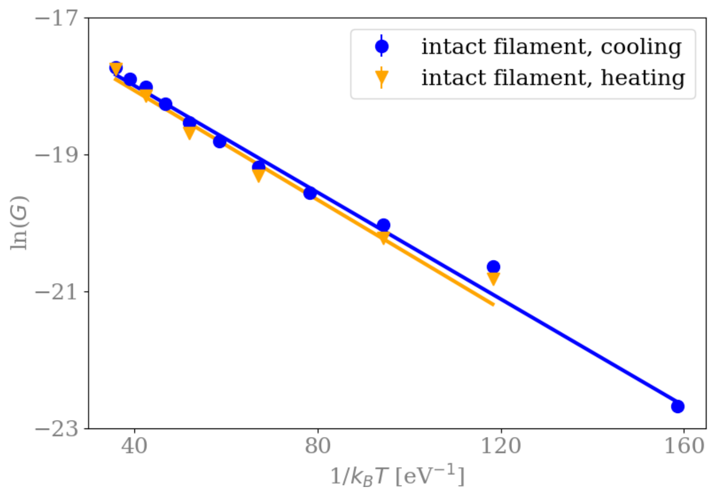

Fig. S6. Cooling and consequent heating of a cable bacterium filament shows the same Arrhenius behaviour with a similar activation energy and hence no decay of the sample.

Table S1. Impedance spectroscopy (IS) fitting results show a small contact resistance. For 7 intact filaments and 4 fibre sheaths, a fit is made with a R(RC)-circuit. Here, R_s the series resistance, R_p the parallel resistance and C_p the parallel capacitance, with R_p showing a large variance in bulk conductivity values. The low value for R_s/R_p indicates the contact resistance is negligible as compared to the bulk resistance.

**Sample**	**Substrate**	**R_s (kΩ)**	**C_p (pF)**	**R_p (MΩ)**	**R_s/R_p (%)**
IS-Intact 1	Glass	0.19	42.35	53.16	0.00035
IS-Intact 2	Glass	0.18	38.42	9.99	0.00176
IS-Intact 3	Glass	0.11	39.87	16.02	0.00071
IS-Intact 4	Glass	0.16	43.47	1301.00	0.00001
IS-Intact 5	SiO2	0.11	49.07	557.90	0.00002
IS-Intact 6	SiO2	1.30	15.34	62.20	0.00209
IS-Sheath 1	SiO2	0.24	50.54	0.87	0.02753
IS-Sheath 2	SiO2	1.57	15.30	27.01	0.00583
IS-Sheath 3	SiO2	3.35	9.03	3645.00	0.00009
IS-Sheath 4	SiO2	7.88	24.54	3.12	0.25219

Table S2. Resistances corresponding to the different combinations of electrodes in Fig. 2A (in MΩ)

**Combination**	**2**	**3**	**4**	**5**
**1**	17.5	39.4	77.5	122.0
**2**		19.8	59.2	104.2
**3**			37.0	82.6
**4**				45.4

Table S3. Mobility calculated for the field effect transistor measurements on four cable bacterium sheaths.

**Sample**	***μ (cm^2 $V^{-1}s^{-1}$)***
FET-Sheath1	0.09
FET-Sheath2	0.11
FET-Sheath3	0.27
FET-Sheath4	0.27

Table S4. Arrhenius analysis for temperature dependent measurements on intact filaments and fibre sheaths shows the activation energy E_a for different substrates.

**Sample**	**Substrate**	**E_a (meV)**
T-DC-Intact1	Au on glass	50.7
T-DC-Intact2	Au on glass	39.0
T-DC-Intact3	Au+CP on glass	37.0
T-DC-Intact4	Au+CP on glass	51.7
T-IS-Intact1	CP on glass	40.8
T-IS-Intact2	CP on glass	34.4
T-IS-Intact3	CP on glass	48.4
T-IS-Intact4	CP on SiO_2	36.6
T-DC-Sheath1	Au on glass	47.2
T-DC-Sheath2	Au+CP on glass	39.1
T-IS-Sheath1	CP on Glass	54.6
T-IS-Sheath2	CP on Glass	55.1
T-IS-Sheath3	CP on Glass	55.1
T-FET-Sheath1	AU+CP on SiO_2	34.4
T-FET-Sheath2	IDE-Au on SiO_2	48.3
T-FET-Sheath3	IDE-Au on SiO_2	38.4
T-FET-Sheath4	IDE-Au on SiO_2	52.1
T-FET-Sheath5	IDE-Au on SiO_2	48.9

Table S5. The temperature dependent impedance results of Fig. 4B were fitted to an (RC) circuit (R_s resistance kept zero) to show R_p follows the DC Arrhenius behaviour and C_p stays constant.

**Temp. (°C)**	**R_p (MΩ)**	**C_p (pF)**
-175	423.8	0.051
-150	176.3	0.0519
-125	94.3	0.0528
-100	57.2	0.0536
-75	39.6	0.0544
-50	29.8	0.0551
-25	23.9	0.0568
0	19.4	0.0576
25	17.6	0.0556
50	19.35	0.0549

Table S6. Activation energy for the mobility and conductivity is calculated for a fibre sheath (n=3) by an Arrhenius fit the range -195 °C to -100 °C

**Sample**	**E_a (meV) from μ**	**E_a (meV) from σ**
T-FET-Sheath2	39.0	48.3
T-FET-Sheath4	29.0	52.1
T-FET-Sheath5	40.1	48.9
